# Pediatric metanephric adenoma with Fanconi–Bickel syndrome: a case report and review of literature

**DOI:** 10.1186/s40792-022-01435-4

**Published:** 2022-05-05

**Authors:** Osama M. Sarhan, Ahmed Al Farhan, Salma Abdallah, Hamzah Al Ghwanmah, Deena Boqari, Helmy Omar, Abdulmohsin Al Faddagh, Hanan Al Kanani, Fouad Al Kawai

**Affiliations:** 1 Urology Department, Faculty of Medicine, Mansoura Urology and Nephrology Center, Mansoura University, Mansoura, Egypt; 2 Urology Department, King Fahad Specialist Hospital, Dammam, 31444 Saudi Arabia; 3 Department of Pediatrics, King Fahad Specialist Hospital, Dammam, Saudi Arabia; 4 Pathology Department, King Fahad Specialist Hospital, Dammam, Saudi Arabia

**Keywords:** Pediatric, Renal mass, Metanephric adenoma, Fanconi–Bickel syndrome, Nephrectomy, Outcome

## Abstract

**Background:**

Metanephric adenoma (MA) is a rare benign renal tumor that resembles renal cell carcinoma and Wilms’ tumor in radiological as well as pathological appearance. It can present at any age or gender, and it is extremely rare in the pediatric age group with less than 50 reported cases. Fanconi–Bickel syndrome (FBS) is a rare autosomal recessive disorder of carbohydrate metabolism. Herein, we report a rare incidence of MA in a boy with a genetically confirmed FBS who underwent a nephron-sparing surgery.

**Case presentation:**

A 21-month-old boy was referred to the pediatric urology clinic for further evaluation of an incidentally discovered left renal mass. His laboratory investigations showed normal renal function, hypophosphatemia, high blood glucose level, markedly elevated serum alkaline phosphatase, and low serum vitamin D. Blood picture showed signs of polycythemia and urinalysis showed glucosuria and aminoaciduria. Genetic testing was positive for Fanconi–Bickel syndrome. Radiological investigations were carried out with abdominal ultrasound and computerized tomography (CT) with intravenous contrast documented a sharply marginated peripheral hypoechoic hypovascular homogeneously enhancing mass at the upper pole of the left kidney measuring 2.0 × 1.8 × 2.0 cm. The child was admitted and started on supportive treatment until his medical condition was stabilized, then underwent elective open left partial nephrectomy via a left upper transverse abdominal transperitoneal incision. The excised renal mass was sent for histopathological assessment and was found to be a tumor composed of tightly packed tubules with no mitotic figures or necrosis and scanty cytoplasm consistent with MA. After good hydration and tumor resection, his polycythemia gradually improved. The patient was discharged home in a good condition with his proper replacement therapies. His follow-up abdominal ultrasound after 12 months showed no signs of recurrence.

**Conclusions:**

Metanephric adenoma is extremely rare in the pediatric age group, especially in those who have a FBS. The only way to diagnose and treat this tumor is by surgical resection as most patients are asymptomatic. A nephron-sparing surgery is better for this age group in which the future renal function is considered.

## Background

Metanephric adenoma (MA) is a rare benign renal tumor that resembles renal cell carcinoma and Wilms’ tumor in radiological and pathological appearance [1–4]. It can present at any age or gender, and it is extremely rare in the pediatric age group with less than 50 reported cases [1, 4, 5]. The diagnosis is only done after surgical resection, and it usually has a favorable prognosis [1, 4].

Fanconi–Bickel Syndrome (FBS) is a rare autosomal recessive disorder of carbohydrate metabolism first described in 1949. It is characterized by nonfunctional glucose transporter 2 (GLUT2) mutation, hepato-renal glycogen accumulation, both fasting hypoglycemia as well as postprandial hyperglycemia and hypergalactosemia indicating an impaired utilization of these two monosaccharides, and proximal renal tubular dysfunction [6, 7]. The typical clinical picture is characterized by hepato- and nephromegaly, rickets, and short stature.

Herein, we report a rare occurrence of MA in a boy with a genetically confirmed FBS who underwent a nephron-sparing surgery (NSS).

## Case presentation

A 21-month-old full-term boy product of normal vaginal delivery, known to have lactose intolerance, has been referred to the pediatric urology clinic for further evaluation of an incidentally discovered left renal mass. His mother noticed a gradually progressive abdominal distension with polyuria, polydipsia, and constipation. The patient's family history includes consanguineous parents with two healthy brothers and ten sisters (2 of them had died during their childhood).

On physical examination, his vital signs were in the normal range, height, and weight on the 25% centile with no dysmorphic features. The abdomen was distended, soft, and lax with no palpable abdominal masses or ascites. Genital examination showed a normal circumcised penis and palpable bilateral testes in the scrotum. In addition, there were positive signs of active rickets.

The initial laboratory workup revealed normal serum creatinine 37 umol/L, reduced serum phosphorus 0.81 mmol/L, markedly elevated serum alkaline phosphatase 2178 unit/L, low serum vitamin D 16.8 ng/ml, elevated blood glucose level 8.9 mmol/L with normal glycosylated HB 5.5%. Additionally, the level of serum lactate dehydrogenase (LDH) was in its normal range (186 unit/L). CBC showed signs of polycythemia (HGB 18.1 g/dL and HCT 52.6%). Urine analysis showed glucosuria and aminoaciduria. Genetic testing was considered because of the family history and clinical presentation, and it was positive for homozygous for the c.474A > C p.(Arg158Ser) familial variant in the SLC2A2 gene, which supported the clinical diagnosis of Fanconi–Bickel syndrome (FBS).

**Fig. 1:**
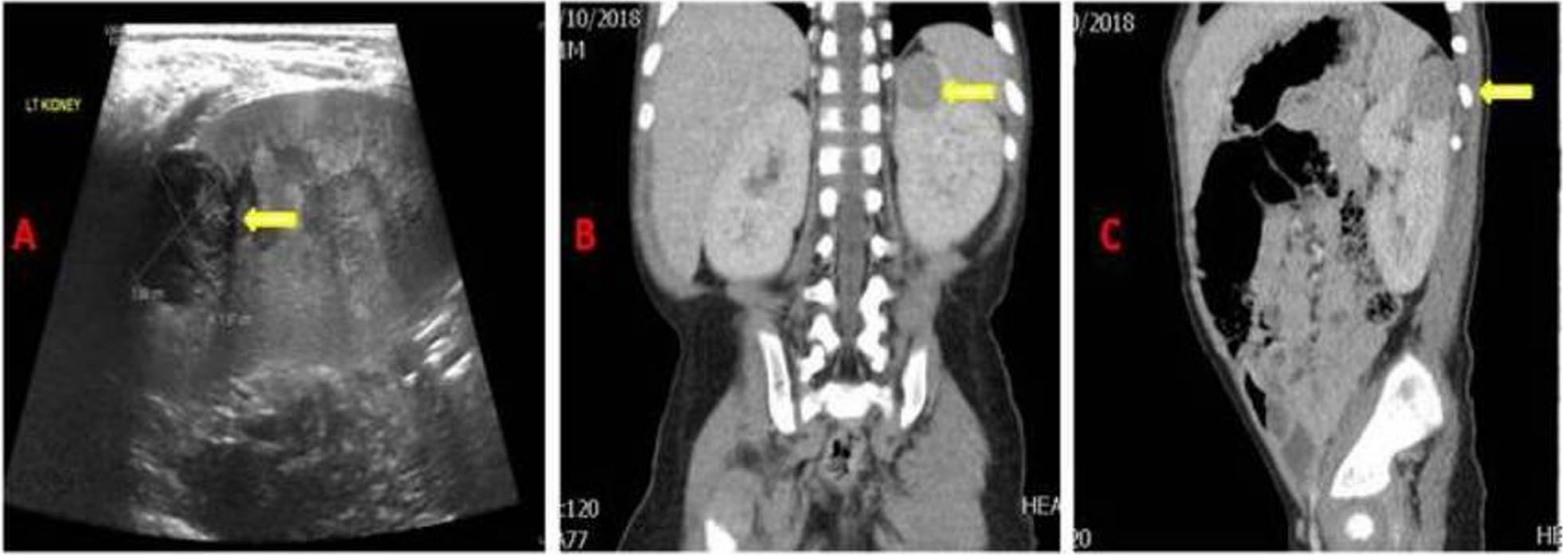
Radiological evaluation. **A** Renal ultrasound documented a sharply marginated peripheral hypoechoic hypovascular mass at the upper pole of the left kidney measuring 2 × 2 cm. **B**, **C** Computerized tomography scan of the abdomen showing a partially exophytic homogenously enhancing mass at the upper pole of the left kidney measuring 1.8 × 1.9 × 2 cm

Radiological investigations were done, and the abdominal ultrasound recognized a sharply marginated peripheral hypoechoic hypovascular mass at the upper pole of the left kidney measuring 2.0 × 1.8 × 2.0 cm (Fig. 1A). Computerized tomography (CT) with intravenous contrast reported a solitary round partially exophytic homogeneously enhancing mass at the upper pole of the left kidney measuring 1.8 × 1.9 × 2 cm in its dimensions. There was no fat density or calcification. The kidneys were mildly enlarged for age and weight. The liver was enlarged measuring 11.3 cm. The anterior ends of the ribs showed splaying and cupping with milder changes in the proximal femoral metaphysis suggestive of active rickets (Fig. 1B, C). After investigation, the child was admitted and started on vitamin D3 and One-Alpha® with sodium phosphate q 6 h with a good response until his alkaline phosphatase was normalized to 350 unit/L and phosphorus to 1.81 mmol/L.

**Fig. 2:**
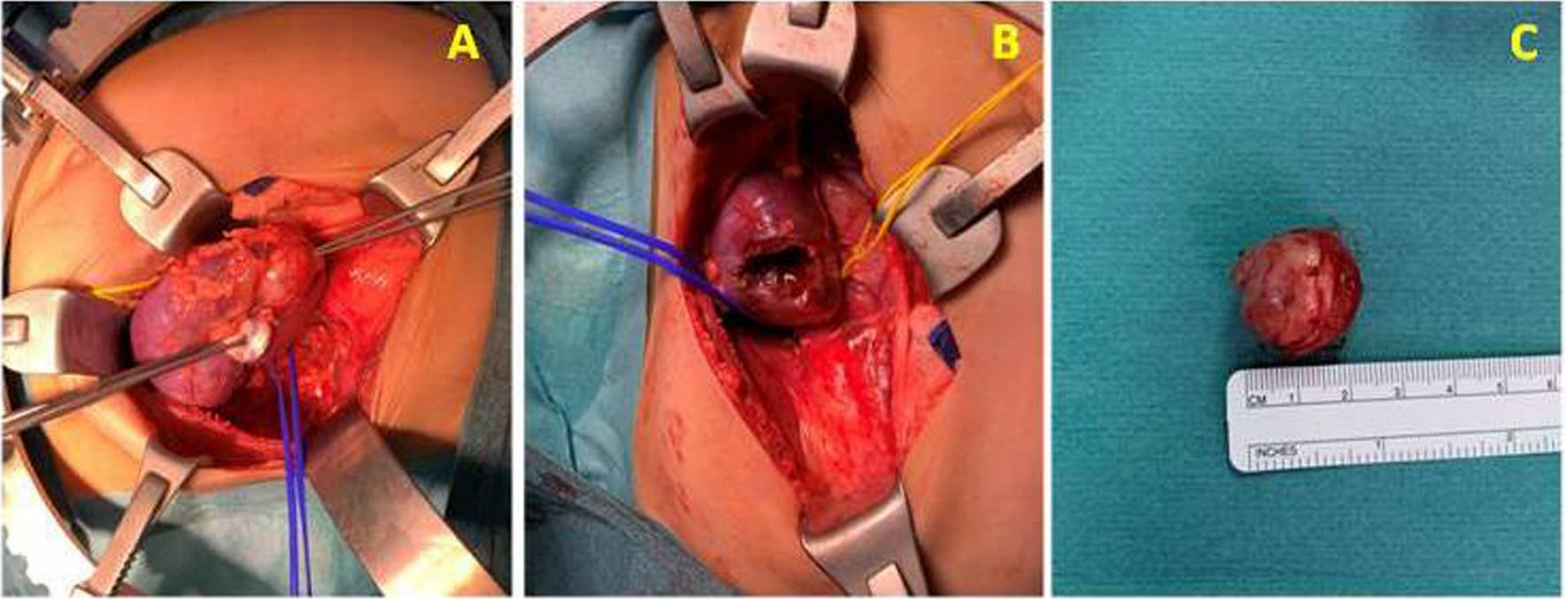
Intra-operative photos. **A** Showing the mass at the upper pole of the left kidney. **B** Showing the left kidney after excision of the mass. **C** Showing the mass after excision

Since the renal mass was small, peripheral, and amenable for resection with no evidence of metastases, and the renal function might be affected by the long-term complications of FBS, a decision of NSS was taken after agreement with his parents. The patient then underwent an elective open left partial nephrectomy via a left upper transverse abdominal transperitoneal incision (Fig. 2).

**Fig. 3:**
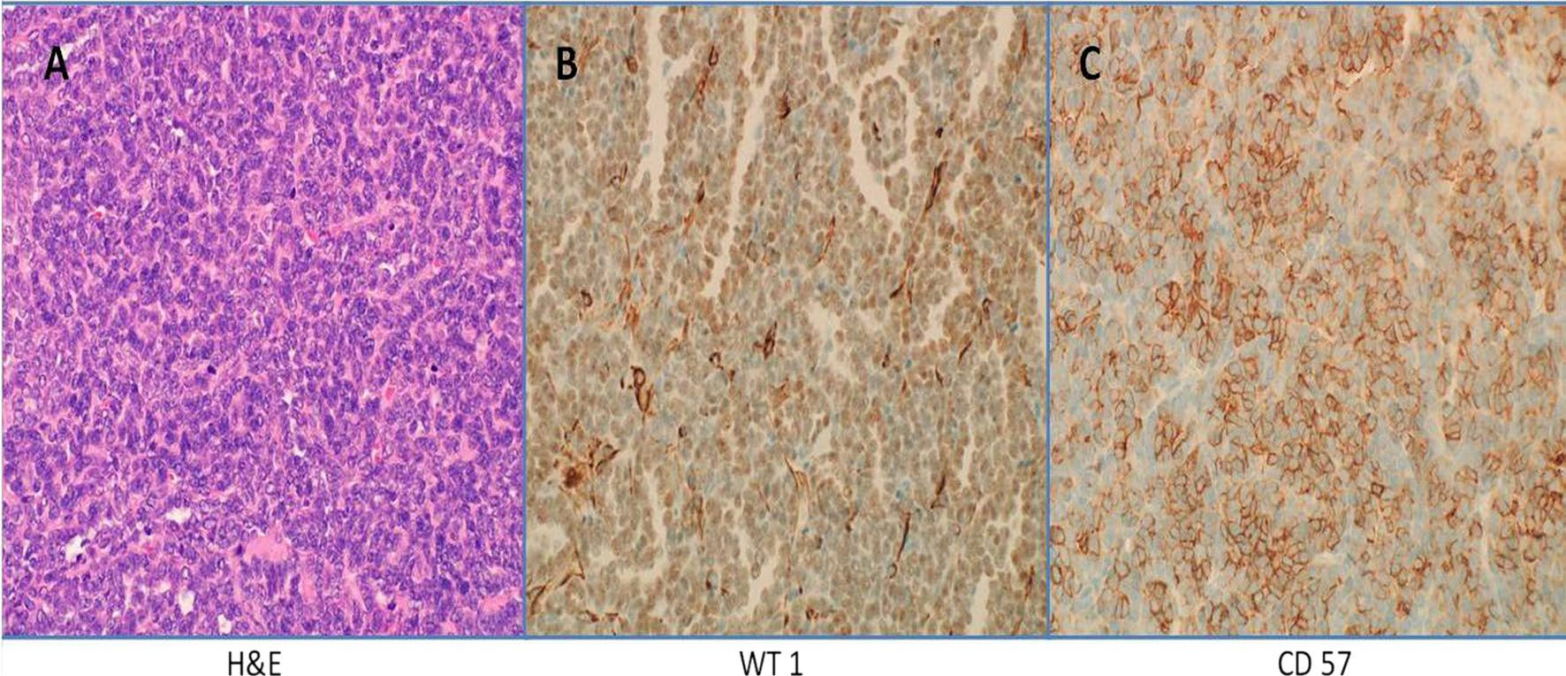
Histopathological examination. **A** H&E stain section from the renal mass excised showing a well-circumscribed tumor composed of tightly packed tubules. The cytoplasm is scanty and the nuclei are small and devoid of nucleoli. No mitotic figures or necrosis is present. **B**, **C** Immunohistochemical staining of the renal mass with WT1 and CD57 showing positive staining

Grossly, the excised tumor was single, firm, and measured 21 × 20 × 18 mm. The cut surface was yellow–white in color. It appeared solid and well-circumscribed with lacked fibrous capsular tissue. The renal mass was sent for histopathological assessment and showed a tumor composed of tightly packed tubules with no mitotic figures or necrosis and scanty cytoplasm consistent with MA. Moreover, Wilms’ tumor and renal cell carcinoma were ruled out by immunohistochemical stains for WT1 and CD57 (Fig. 3).

After good hydration and tumor resection, his polycythemia began to improve, and he maintained his HGB around 13 g/dL. The patient was discharged home in a good condition with his proper replacement therapies. His follow-up abdominal ultrasound after 12 months showed no signs of recurrence. The patient is stable now and has no active complaints, but he needs further follow-up for any new events.

## Discussion

Metanephric adenoma is a kind of tumor that is rarely found in pediatric patients and has also been called a renal epithelial tumor, nephrogenic nephroma, and embryonal adenoma [1–5, 8]. Meanwhile, Fanconi–Bickel syndrome is a rare autosomal recessive disorder characterized by nonfunctional GLUT2 mutation, hepato-renal glycogen accumulation, both fasting hypoglycemia as well as postprandial hyperglycemia, and hypergalactosemia indicating an impaired utilization of these two monosaccharides, and proximal renal tubular dysfunction [6, 7]. The association between the two pathologies is extremely rare; hence reporting this case in pediatrics gave this report its scarcity.

The age range of patients presenting with MA varies widely from 15 months to 83 years [1, 4, 9]. Metanephric adenoma is reported to be more common in females, and tumors as small as 0.3 cm to as large as 15 cm have been described. In the context of MA, the developmental abnormality of other body systems has not been presented [1, 4]. However, association with other genetic syndromes is expected as MA was linked with some genetic mutations [10, 11].

FBS is an extremely rare disease, but with a distinct clinical entity. It usually presents below the age of 1 year with failure to thrive, severe hypophosphatemic rickets, and hepatomegaly [6, 7]. Proximal renal tubular dysfunction with glucosuria, phosphaturia, generalized aminoaciduria, bicarbonate wasting, and hypophosphatemia are characteristic findings [6, 7]. By two years of age, enlarged kidneys are noticed clinically. There have been no previous reports about any association with renal tumors. To our knowledge, this is the first case in the literature of a FBS infant with MA.

Our case was incidentally discovered, and most of the MA cases have no symptoms. Abdominal, flank pain, or hematuria could be other modes of presentation [1, 4, 5, 9]. Polycythemia and MA are frequently associated with 12% and 30% incidences in adults and children, respectively [1, 4, 8]. Yoshioka et al. in 2007 documented that MA can produce erythropoietin and hence cause polycythemia [12]. Our case of MA was associated with polycythemia and the blood picture was improved after surgical resection.

It is hard to discriminate between MA and other renal tumors at presentation [3]. Differential diagnosis includes Wilms’ tumor, other malignant tumors of the kidney such as clear cell sarcoma or rhabdoid tumors, or even renal cell carcinoma. Various imaging techniques cannot reliably differentiate between the benign from malignant nature of MA. There are certain imaging features that can help to suspect MA on renal ultrasound and CT scans [3, 4, 9, 11]. MA is usually hyperechoic and well-circumscribed on ultrasound and with lower attenuation than enhancing renal parenchyma on CT [4, 5, 8].

On a histopathological level, MA is composed exclusively of epithelial elements with tubular, glomeruloid, or papillary architecture, a virtual absence of mitoses and nucleoli, no vascular involvement, and negativity for cytokeratin 7 (CK7) and epithelial membrane antigen (EMA). The cells have rounded nuclei with scanty cytoplasm and low mitotic activity. These findings were consistent with our case in addition to the positive WT1 and CD57 staining [9, 10].

Surgical excision is the mainstay of management of MA because of its unknown malignant potentiality. Both radical and partial nephrectomies were reported either by open or laparoscopic approach [3–5, 11, 13, 14]. The decision depends mainly on the size and location of the tumor and its potential malignant nature. The renal function was reported to be stable or slightly decreased over time. Several reports supported the benefits of performing NSS for MA but most of these reports concerned adult patients and only a few cases were pediatric [3, 4, 13, 14].

In general, a solid renal mass found in a 21-month-old child is most likely to be a Wilms’ tumor. Based on the current guidelines, radical nephrectomy is the standard treatment, but recent data supported the efficacy of NSS in treating Wilms’ tumors even in those without tumor-associated syndromes such as Beckwith–Wiedemann syndrome and Denys–Drash syndrome [15–17]. In addition, renal dysfunction is predicted in the future due to the complication of FBS. For that reason, a NSS was performed in our case to preserve the functioning renal parenchyma.

After surgical resection of the tumor, no specific therapy is recommended. There have been reports of MA metastases, even though they are considered benign [2, 18]. Relapse and mortality did not occur in the reviewed literature on pediatric MA [3, 4]. There are no clear guidelines for the follow-up of such cases. Only a physical examination with imaging on a regular basis was recommended with no clear expectations regarding its outcome.

Finally, we think that the combination of MA and FBS in our case was a mere coincidence. To our knowledge, there was no reported association between FBS and childhood tumors except for one report of hepatocellular carcinoma [19]. GLUT gene family has a role in cancer metabolism and cancer targeted therapy and GLUT 2 has been expressed in some cancer cell lines indicating that it is involved in the cancer metabolism, not in carcinogenesis. Due to the rarity of the disease, we believe that there is no clear consensus for periodic screening of malignancies in patients with FBS.

## Conclusions

Metanephric adenoma is very rare in the pediatric age group, especially in those diagnosed with FBS. The only way to diagnose and treat this tumor is by surgical resection as most patients are asymptomatic. A NSS is a valid option in this age group in which the future renal function is considered. It will be of great benefit to find a less invasive and accurate diagnostic method for this tumor to conduct a more conservative management protocol.

## Data Availability

Not applicable.

## Abbreviations

MA: Metanephric adenoma
FBS: Fanconi–Bickel syndrome
NSS: Nephron-sparing surgery
